# A future location prediction method based on lightweight LSTM with hyperparamater optimization

**DOI:** 10.1038/s41598-023-44166-8

**Published:** 2023-10-20

**Authors:** Ha Yoon Song

**Affiliations:** Department of Computer Engineering, Hongik University, 72-1 Sangsu, Mapo, Seoul, 04066 Korea

**Keywords:** Engineering, Mathematics and computing

## Abstract

In this study, we presented a method for future location prediction based on machine learning over geopositioning data sets. There are large amounts of geopositioning data sets collected by mobile devices mainly due to modern geopositioning systems such as GPS, GLONASS and Galileo. Based on these geopositioning data sets, it is possible to have a wide variety of location-based services. These data sets can be used for future location prediction of objects, especially humans. Additionally, they have a high possibility for further applications. The purpose of this research is to present a simple and lightweight method that can be applicable to devices with lower computing capability devices, such as AIoT (Artificial Intelligence of Things) or EdgeML (Edge Machine Learning) devices. We introduced a basic LSTM (Long Short Term Memory) model with hyperparameter optimization, especially on window size of continuous geopositioning data, using limited previous geopositioning data for location prediction purposes. We found that the results of using our method for future location prediction are considerably fast and accurate compared with existing neural network-model-based approaches. We also applied our method to non-continuous geopositioning data sets and found it to be equally effective.

## Introduction

Geopositioning data can now be collected through a variety of methods, resulting in their active use by location based services^1^. This study proposes a technique for predicting users’ near future locations in real time by learning their previous movement trajectories. Devices such as AIoT (Artificial Intelligence of Things)^2^ and EdgeML (Edge Machine Learning) devices^3^ typically run applications with lower computational power requirements compared with powerful computer systems for machine learning. To accomplish this, the simplest algorithms possible should be used, and the time required to process data should be minimized. Location data were collected from smartphones and IoT (Internet of Things) devices. A total of 17 people’s trajectory data were collected every 2 s using smart phones and IoT devices to create the location data set used in this study. Although the data set contains several distinct characteristics, only three data characteristics, [Latitude, Longitude, Time] required for learning, were extracted and pre-processed. Preprocessed data were used for location data pattern analysis and future locations were predicted using the analyzed contents. The LSTM (Long Short Term Memory)^4^ model was used for training because the trajectory data is time series data. The remainder of this paper is organized as follows. Section “Related work” reviews studies related to this research. Section “Features on data sets” describes the preprocessing process after selecting a data set, based on whether or not intermediate data is lost among the collected data. The Section “Hyperparameter optimization on window size” discusses how to optimize hyperparameters for different models. Because the structure of each model differs, the optimal hyperparameters tuning per model must be determined. The experimental process and its outcomes are discussed in Section “Experimental results”. The experiment used three types of data sets: seven data sets with continuous trajectories are grouped as C Type data sets; six data sets with non-continuous trajectories are named N type data sets; four data sets with less than 100 data counts are termed L type data sets. The learning model was LSTM, and each model’s hyperparameters were tuned using a hyperparameter optimization method. Furthermore, movement data was visualized after being predicted using existing movement data and learning. In Section “Treating non-continuous data sets”, we applied a simple method to deduce C type data set from an N type data set using the concept of course data. Then, we applied our location prediction method to course data and our method was found to be effective with the N type data set. Section “Conclusion and future works” discusses the significance of the findings of this study as well as future research directions.

## Related work

There are previous research on location prediction using various techniques. The existing techniques^5^ used to predict location are broadly classified into two types: convolutional neural network (CNN) and recurrent neural network (RNN). The CNN-based techniques make predictions by visualizing trajectory data. The preprocessing of trajectory data to visualize it is a complicated process. First, the dimension of [Latitude, Longitude] data must be reduced to [Label] value using area partitioning. This is done to reduce the amount of calculation; however, when the dimension is reduced, the relationship between the location data is destroyed when compared to using the original data [Latitude, Longitude]. The predictions are based on the following ideas.

**Table 1:** Example of mobility trace preprocessing.

Trajectory	Predict
(1,0,0,0,0,0)	2
(1,2,0,0,0,0)	3
(1,2,3,0,0,0)	4
(1,2,3,4,0,0)	5
(1,2,3,4,5,0)	6
(1,2,3,4,5,6)	0

Table 1 shows the process described in^5^. The process partially attaches the trajectory data to visualize the (1,2,3,4,5,6) trajectory. Given trajectory data (1,2,3,4,5,6), for example, (1,0,0,0,0,0) is appended to the first trajectory and 2 is appended to the predicted value. The remaining trajectories are handled in the same manner. By appending 0 to the predicted value, the final trajectory is indicated. The trajectory data is visualized in this way, and the predicted values are obtained. At this point, the predicted value also yields a label value; however, there is some disparity with the original [Latitude, Longitude] data. Therefore, the RNN-based method, which directly processes [Latitude, Longitude] data, was used in this study. Because the RNN method uses [Latitude, Longitude] data directly, there is no need for partially pasting the trajectory. Hence, prediction values with RNNs are more intuitive to interpret than with CNNs. Recurrent neural network learning continues by using previously learned data as the next correct answer. Therefore, it is appropriate for data with time series and dynamic characteristics. However, the vanishing phenomenon^6^ is unavoidable when learning with short-term memory. We used an LSTM rather than a simple RNN in our study because we needed to predict future data using not only previous data but also macroscopically past data. Through this, the vanishing phenomenon was solved^7^. Rather than simply remembering previous data, the LSTM model enables their memorization more macroscopically. The number of previous data points to be remembered is defined as Window_Size. Optimizing the hyperparameter value is therefore required. Related issues are discussed in detail in Section “Hyperparameter optimization on window size”. There were other research that used LSTM: ‘Social LSTM’^8^, which considers the influence of other people around the experimenter, using multiple LSTM models; trajectory prediction using attention network, which is frequently used in natural language processing^9^; trajectory prediction using basic LSTM^10^. There is a method that uses the LSTM weighted values of people around a subject to make a social pool, assuming that the movements of people around it affect the subject, and then applies it to learning^8^. When this model is used, the average displacement error (ADE) and final displacement error (FDE) value is found to be 0.27 km and 0.61 km respectively; ADE refers to the mean square error (MSE) over all estimated points of every trajectory and the true points; FDE means the distance between the predicted and true final destinations. Another research predicts a trajectory using an attention network^9^. The attention network is primarily used for natural language processing; it differs from the LSTM in that the LSTM can control how much information from the past is remembered, whereas the attention network remembers everything from the past. When this model is used, the ADE and FDE value is 0.50 km, and 1.06 km, respectively. Yet another method employs the default LSTM model^10^, which predicts a trajectory by defining look_back as the amount of remembered past data and adjusting look_back to the values 10, 20, and 30. The learning time was 40.8642 s when look_back was 10 and 51.6841 s when it was 30, and it varied widely depending on the look_back value. In this study, we present an *LSTM with an optimized Window_Size hyperparameter* that optimizes it after defining the amount of past data to be stored as Window_Size. Here, we discovered that when Window_Size is optimized for each data set, learning time and accuracy are optimized. This resulted in more accurate ADE and FDE values than when other models were added to the LSTM^8^^9^, as well as the advantage of significantly shortening the learning time when compared with simply applying the LSTM^10^.

## Features on data sets

**Table 2:** Typical characteristics of collected geopositioning data sets.

Data set ID	Number of data	Continuity of collection
0	1029	N
1	263	C
2	209	C
3	370	N
4	537	C
5	1	L
6	157	N
7	523	N
8	990	C
9	139	C
10	350	C
11	160	N
12	51	L
13	344	N
14	0	L
15	95	L
16	112	C

Typical characteristics of geopositioning data used in this study is shown in Table 2. Data sets contain trajectory information for 17 people. If the data count is less than 100, the data set is regarded as not applicable. The collected data are divided into seven or six data sets depending on whether or not some part of data was lost in the middle, i. e., depending on the continuity of the geopositioning data. Data types are classified as one of [C,N,L]; C stands for ‘continuous,’ indicating a complete data set; N stands for ‘Noncontinuous,’ indicating a data set with data loss inside the trajectory of data; and L stands for ‘Lack of Data,’ indicating a data set with 100 or fewer geopositions in the data set. C type data sets were [1,2,4,8,9,10,16], N type data sets were [0,3,6,7,11,13], and L type data sets were [5,12,14,15]. The data were divided into three categories: training, validation, and test data, in the ratio of 6:2:2^11^. The preprocessed location data consists of [Latitude, Longitude, Time]. Trajectory data is information in which location data is arranged in ascending order by time. Therefore, after sorting in ascending order based on time, only [Latitude, Longitude] were extracted and preprocessed for calculation purposes. In other words, mainly two properties in the preprocessed data set are used. Machine learning may not be effective if latitude and longitude data are used directly for training because the scale of the data is different. The data range was therefore scaled in this study using MinMaxScaler^12^.

**Table 3:** Sliced data set according to window.

	lat	lon	shift_1_lat	shift_1_lon	shift_2_lat	shift_2_lon	shift_3_lat	shift_3_lon
4	0.045822	0.982501	0.036837	0.987885	0.021883	0.991520	0.010482	0.995558
5	0.057203	0.978732	0.045822	0.982501	0.036837	0.987885	0.021883	0.991520
6	0.070380	0.976847	0.057203	0.978732	0.045822	0.982501	0.036837	0.987885
…	…	…	…	…	…	…	…	…
155	0.992812	0.015345	0.985924	0.022345	0.978137	0.029748	0.970650	0.037017
156	0.998802	0.007403	0.992812	0.015345	0.985924	0.022345	0.978137	0.029748
157	1.000000	0.000000	0.998802	0.007403	0.992812	0.015345	0.985924	0.022345

The inputs for the LSTM model are in the form of three-dimensional values: Number of Samples, Window_Size, and Number of Features. After normalizing the data, Table 3 is created with the Window_Size set to 3 and the input dimension converted to three dimensions. Table 3 shows sequence data that stores the previous three [Latitude, Longitude] values. Here, [Latitude, Longitude] is set to [lat, lon] for convenience of notation. There are lat and lon values in the column value, as well as shift_N_lon and shift_N_lat values that have been moved N times. Because the Window_Size is 3, we can see that it begins on line 4. Using line 4 as an example, the values in lat and lon represent the fourth position data, shift_1_lat, shift_1_lon represents the third position data, and shift_2_lat, shift_2_lon represents the second position data. shift_3_lat, shift_3_lon represents the first position data. In this way, a sequence containing the previous three [lat, lon] values was created.

## Hyperparameter optimization on window size

Because learning is performed for each trajectory data, the process of hyperparameter optimization is required^13^. The LSTM model used in this study is a model that makes predictions by remembering information from the past. As a result, determining how much information from the past will be remembered is crucial^4^. The we call the hyperparameter as Window_Size, standing that how many past geopositioning data are stored for future location prediction. Four methods are typically used to optimize hyperparameters. ‘Manual Search’ is based on people’s intuition or experience, ‘Grid Search’ determines the range of hyperparameters to be searched and searches by substituting values at regular intervals, ‘Random Search’ determines the range of hyperparameters and finds optimal values at random, and ‘Bayesian Optimization’ finds an optimal solution that maximizes an objective function based on Bayes theorem.

The parameter to be obtained in this study is Window_Size. Grid search method was used because it was determined that a simple grid search could be used to obtain it very effectively and efficiently. For the experiment, the Window_Size range was set to 2 to 20. The reason for starting the range at 2 is that starting at 1 is equivalent to using the data source as is, therefore, it started at 2 and ended at 20 because the ADE value tends to increase as Window_Size increases. From our initial experiments to determine the range of Window_Size, Window_Size more than 20 shows meaningless results. After determining the range of hyperparameters to be searched from 2 to 20, optimization was performed using ‘grid search’ to find the optimal value at intervals of 1. Train and validation data were used for evaluation^14^, and the Window_Size for the best performance was extracted.
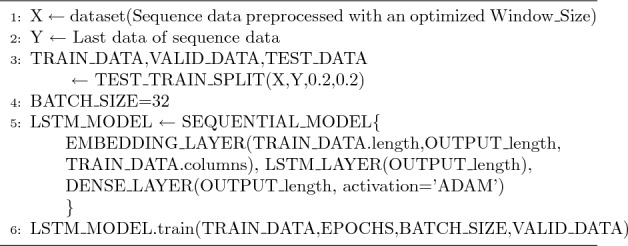


Algorithm 1 is the process of learning LSTM_MODEL. Algorithm 1 is the LSTM algorithm used in Algorithms 2 and 3. X is the formatted sequence data that is preprocessed, as shown in Table 3, and Y is the preprocessed final data. In a 6:2:2 ratio, the data is divided into training, validation, and test data. When training the LSTM model, training and validation data are used. When creating the LSTM model, the output value was set to OUTPUT_length. Because the format of OUTPUT is [Lon, Lat], length is 2. Therefore, when training, two values are produced as outputs, i.e., [Lon, Lat] values. Adaptive moment estimation (ADAM) was used as the activation function.
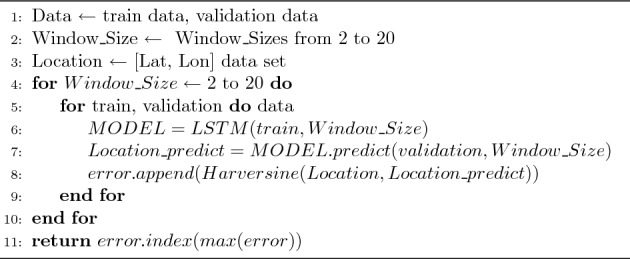


An algorithm 2 is a code that optimizes the hyperparameter Window_Size. As Window_Size increases from 2 to 20, the Harversine values^15^ of the values that appear when the model is trained with training data and validated with Algorithm 1 are stored in an error list. When all training is completed, the index of the minimum value of the error list is returned, as well as the Window_Size value that generates the minimum value of the error, i.e., the Optimized_Window_Size value.

**Figure 1:**
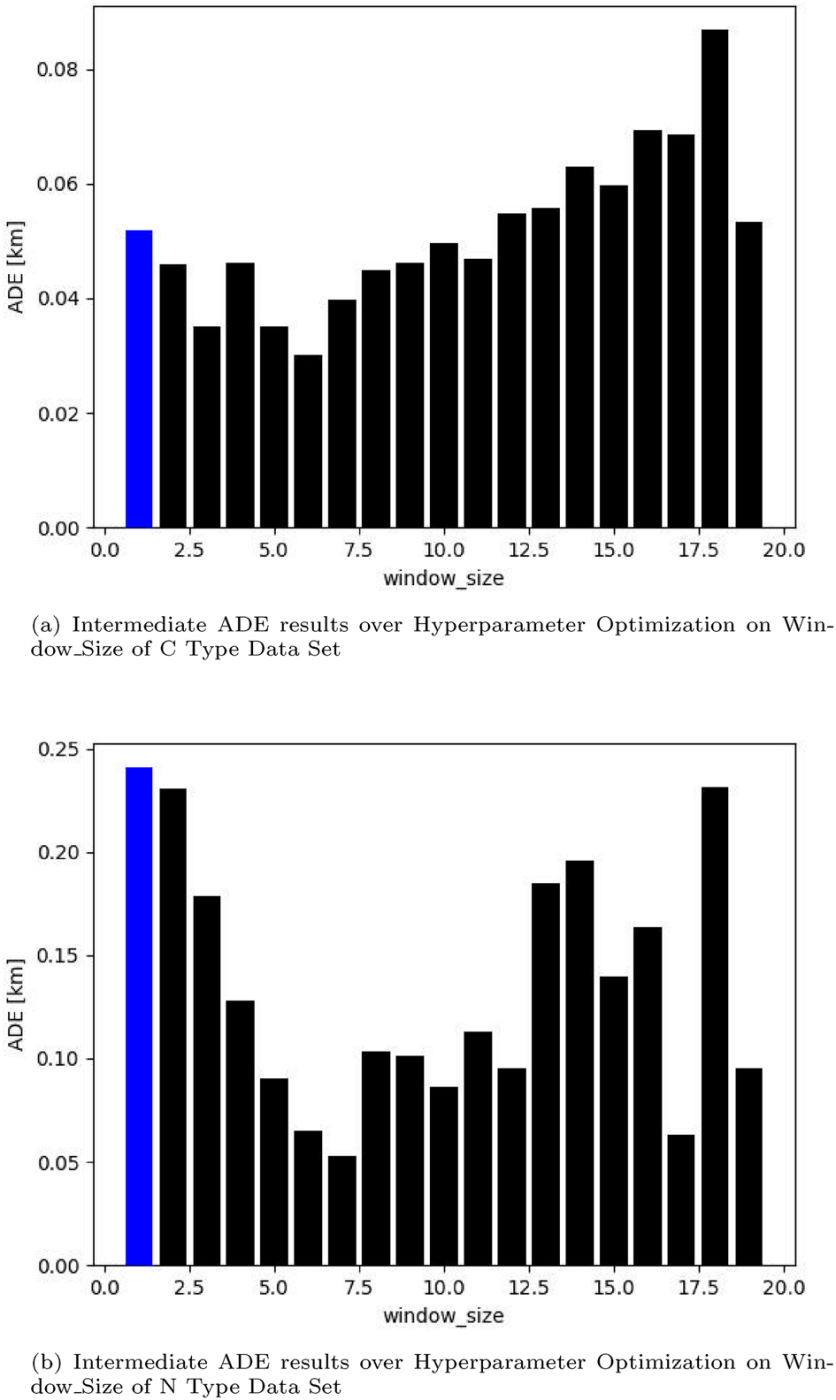
Typical values of hyperparameter optimization w.r.t. C type data set and N type data set.

Figure 1a shows the result of the ADE values over Window_Size for C type Data Set 1, and Fig. 1b shows the result of the ADE values over Window_Size for N type data set 7. The leftmost blue bars represent the outcome when the Window_Size is 1. The implication of this result is that optimization of window size is essential for each data set because the results are greatly dependent on the data count and the distribution of data. As expected, C type data sets have lower ADE values compared with N type data sets. Additionally, for N type data set, which has discontinued parts, the ADE values are equally dependent on Window_Size. From these results, continuity of geopositioning data affects the ADE value of predictions; hence, we will consider C type data sets separately from N type data sets. For N type data sets, we will extend our method in Section “Treating non-continuous data sets”. Hereafter, the unit for ADE and FDE values in tables is in meters, abbreviated as m, as we found most of ADE and FDE values is less than 1 km.

**Figure 2:**
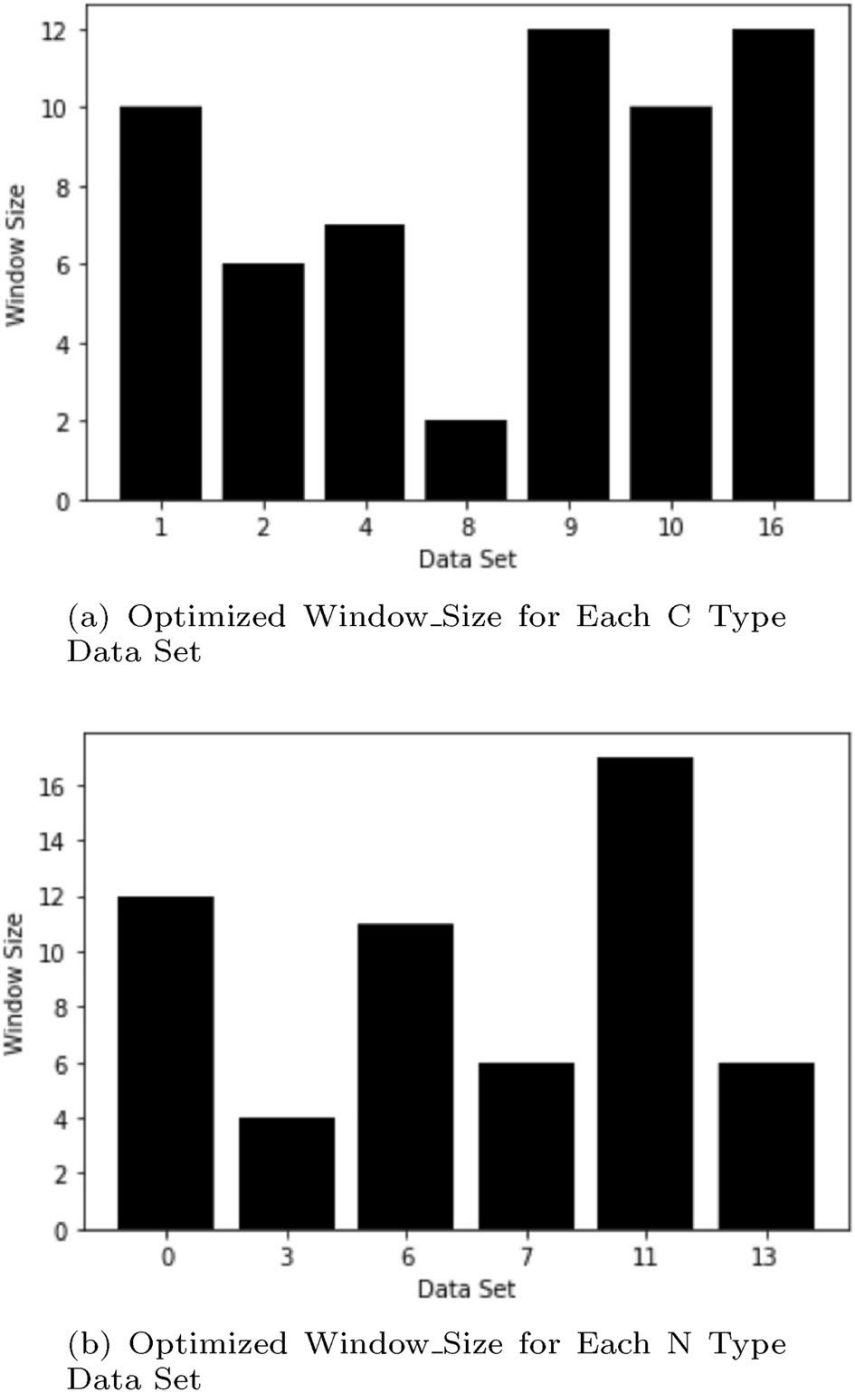
Window_Size identified for C type data set and N type data set.

The results in Fig. 2 show the optimized window size values identified over all the data sets.

## Experimental results

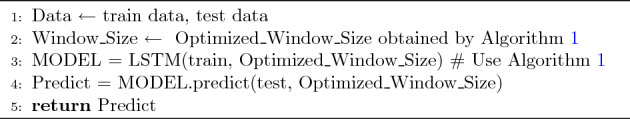



Time and performance were the determining factors in choosing the model to be employed in this study. The training period was shortened, and accuracy of prediction was adequate in our method. Algorithm 3 finds the Optimized_Window_Size using Algorithm 2. Using this value, the LSTM model is trained with training data, and then the prediction value is extracted using the test data. The appraisal of experimental data is based on a number of criteria. To measure prediction accuracy, two markers were employed: The difference between the actual final location and the predicted final position is known as the FDE^10^, while the ADE^9^ is the difference between all actual positions and the positions that were predicted. Note that there are two different ADE values: for Hyperparameter Optimization used in Algorithm 2 and for evaluation of prediction accuracy used in Algorithm 3. One of the methods we used for estimating the distance between two geopositioning data is the Harversine formula^15,16^.

**Table 4:** Summarized results for C type data sets.

Data set ID	FDE (m)	ADE (m)	Training time (s)	Window_Size	Number of data
1	56.578	58.606	7.459214	9	263
2	13.515	21.465	8.530682	6	209
4	135.234	109.991	14.810614	7	537
8	90.524	73.281	64.190169	2	990
9	51.490	40.714	8.243129	12	139
10	91.498	158.801	14.457857	10	350
16	24.437	36.210	6.678380	12	112

**Table 5:** Summarized results for N type data sets.

Data set ID	FDE (m)	ADE (m)	Training time (s)	Window_Size	Number of data
0	339.423	374.328	18.205739	11	1029
3	953.128	927.367	7.532523	4	370
6	259.791	261.752	6.347854	10	157
7	110.007	167.796	8.200427	5	523
11	566.903	430.447	8.945682	15	160
13	1285.301	1407.504	23.193552	3	344

**Table 6:** Numerical results of C type and N type data sets.

	Continuous data set	Noncontinuous data set
FDE	66.182 m	585.759 m
ADE	71.295 m	594.866 m
Average training time	17.767149 s	12.070963 s
Window_Size	Optimized_Window_Size	Optimized_Window_Size

For the experiments, we used Intel(R) Xeon(R) CPU @ 2.20 GHz provided by Google Collaboratory (Colab)^17^. Tables 4 and 5 display the results for C type and N type data sets, respectively. In these instances, the hyperparameter-optimized Optimized_Window_Size value for the data set was applied.

Each table shows the following information: data set, FDE, ADE, training_time, Window_Size, data_size. Window_Size is optimized so that the ADE value is minimized. Table 6 shows the FDE, ADE, Average Training Time, and Window_Size values of C type and N type data sets. Evidently, C type data sets have smaller error values compared with N type data sets. For example, Table 6 shows the FDE and ADE values of C type data sets average 66 m and 71 m respectively, while N type data sets average 586 m of FDE and 594 m of ADE respectively. Average training time identified for C type data sets is 17.7671 s and 12.0709 s for N type data sets.

### Further considerations on window size

As already mentioned, C type data sets have a Window_Size value as 2 in the case of Data Set 8. Logically, a Window_Size of 2 requires more training time because it handles only two geopositioning data as a unit of prediction. For each execution, ADE values vary within a range due to inherited randomness in hyperparameter optimization and LSTM even with the same Window_Size. And thus, Windows_Size for a data set varies according to similar ADE values within error range. For example, Data Set 4 shows similar ADE values, around 0.030, in hyperparameter optimization stage for different Window_Size. Once we have similar ADE across different Window_Size, then we need to choose window size with smaller training time. To investigate this situation further, we tested for various window size values as an additional experiment.

**Figure 3:**
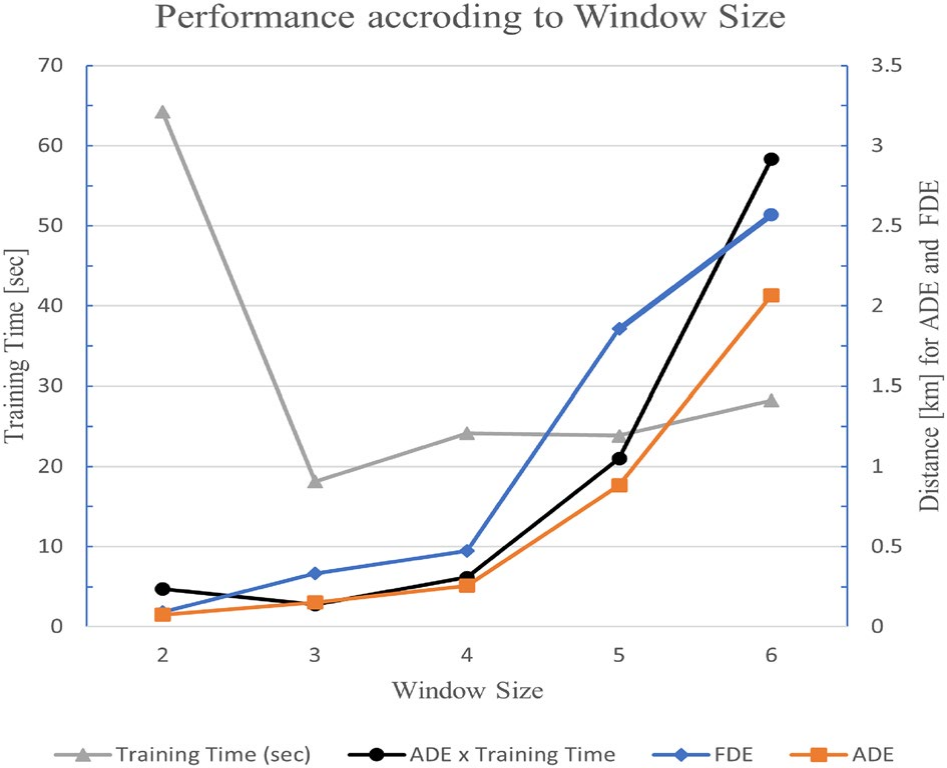
Performance according to window size for data set 8.

Figure 3 shows performance measures according to window size values for Data Set 8, which has optimized window size 2 and therefore has the highest training time. The left y axis is for training time in seconds, and the right y axis is for ADE and FDE in kilometers. ADE $\times$ Training Time is also shown in the graph to show the tendency. The x axis is for Window_Size. Training time varies drastically from Window_Size of 2 to 3. The ADE and FDE values also vary drastically from Window_Size of 3 to 5. Our object function is to minimized ADE and FDE as well as minimize the training time. Therefore, we used a metric of ADE $\times$ Training Time with respect to Window_Size.

**Table 7:** Performance change on C type data set 8.

Window_Size	FDE (m)	ADE (m)	Training time (s)	ADE $\times$ Training time
2	90.524	73.281	64.190169	4.703920
3	331.411	153.063	18.094606	2.769615
4	474.310	254.558	24.163500	6.151032
5	1855.934	880.896	23.841432	21.001827
6	2566.395	2065.696	28.230093	58.314814

Table 7 shows corresponding values of Fig. 3 and we identified the smallest ADE $\times$ Training Time value with Window_Size of 3. Table 7 shows Window_Size corresponding to the smallest ADE value. Training time is also affected by Window_Size and the distribution of geopositioning data. Therefore, it is better to set Window_Size as 3 for Data Set 8 once training time is concerned.

Once we have similar ADE values for hyperparameter optimization for different values of Window_Size, it is possible to select criteria of ADE $\times$ Training Time. However, Window_Size could be selected with optimal ADE if minimizing errors is a main concern.

**Figure 4:**
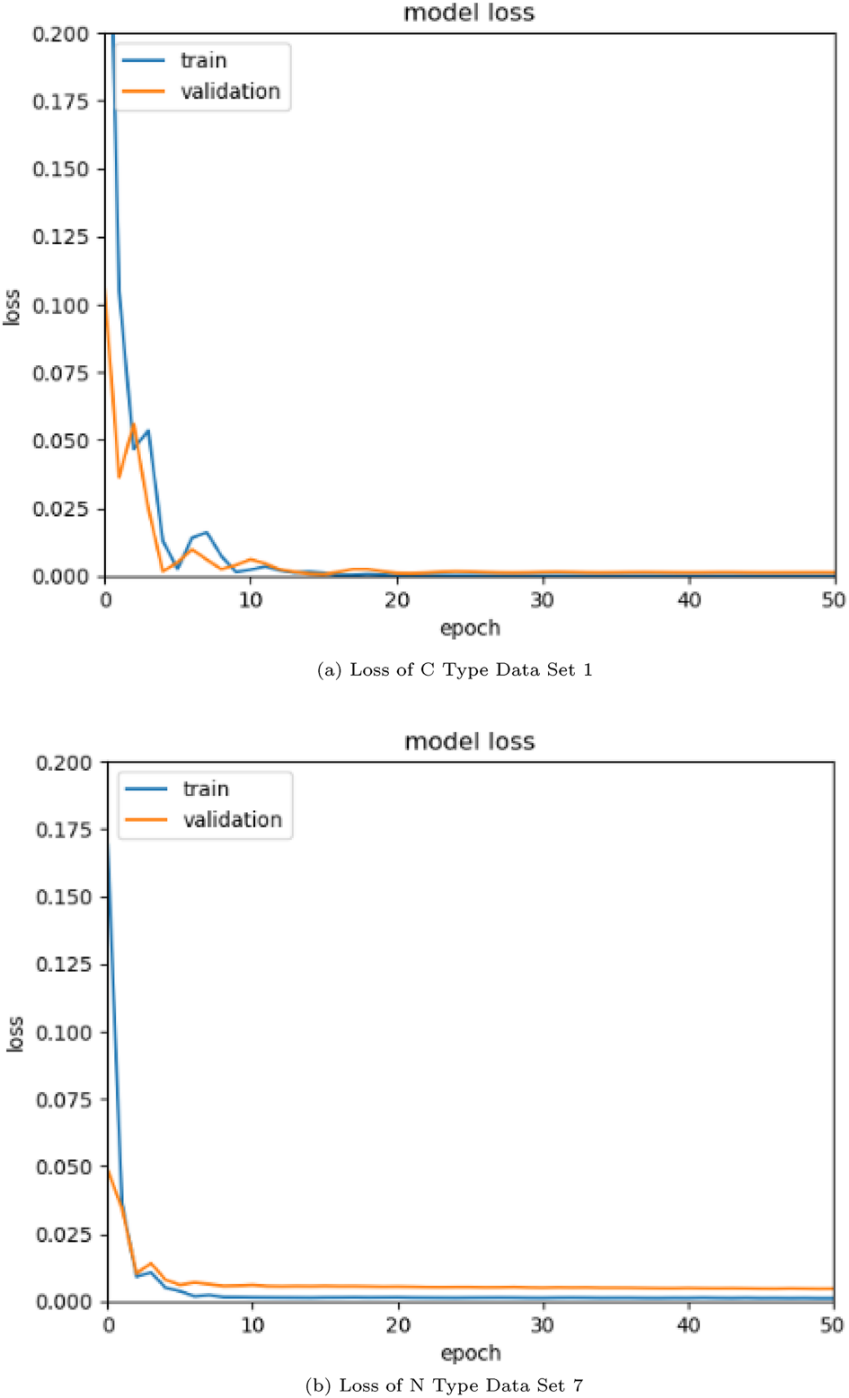
Typical loss of C type and N type data set.

Figure 4a shows loss of Data Set 1 belonging to C type data sets, and Fig. 4b is a graph of loss values according to the epoch of Data Set 7 belonging to N type data sets. In general, for learning to be effective, the gap between the loss of training data set and the loss of validation data set should narrow as the epoch grows. When the epoch increases, the difference is still present in Fig. 4b, unlike in Fig. 4a, where it nearly completely vanishes by epoch 20. This demonstrates that the N type data set requires a long time to learn and has poor accuracy. Conversely, the C type data set exhibits rapid learning and better precision. We can see from this that it is crucial to avoid losses while collecting data, and a mechanism is required to fill it in if data loss occurs.

**Figure 5:**
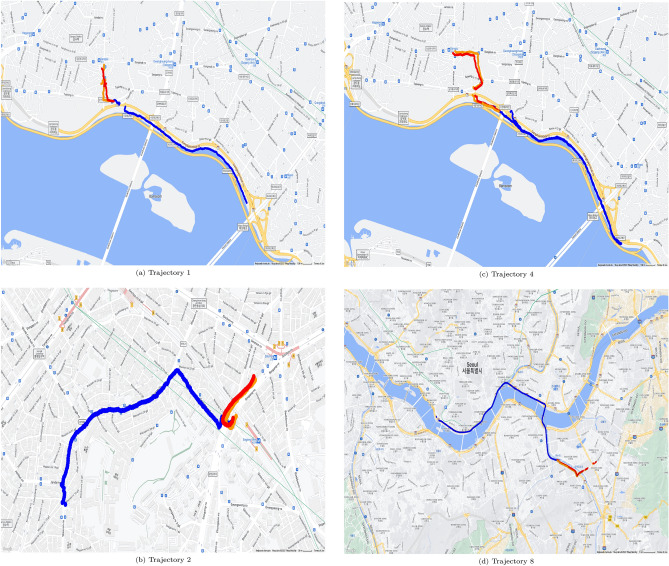

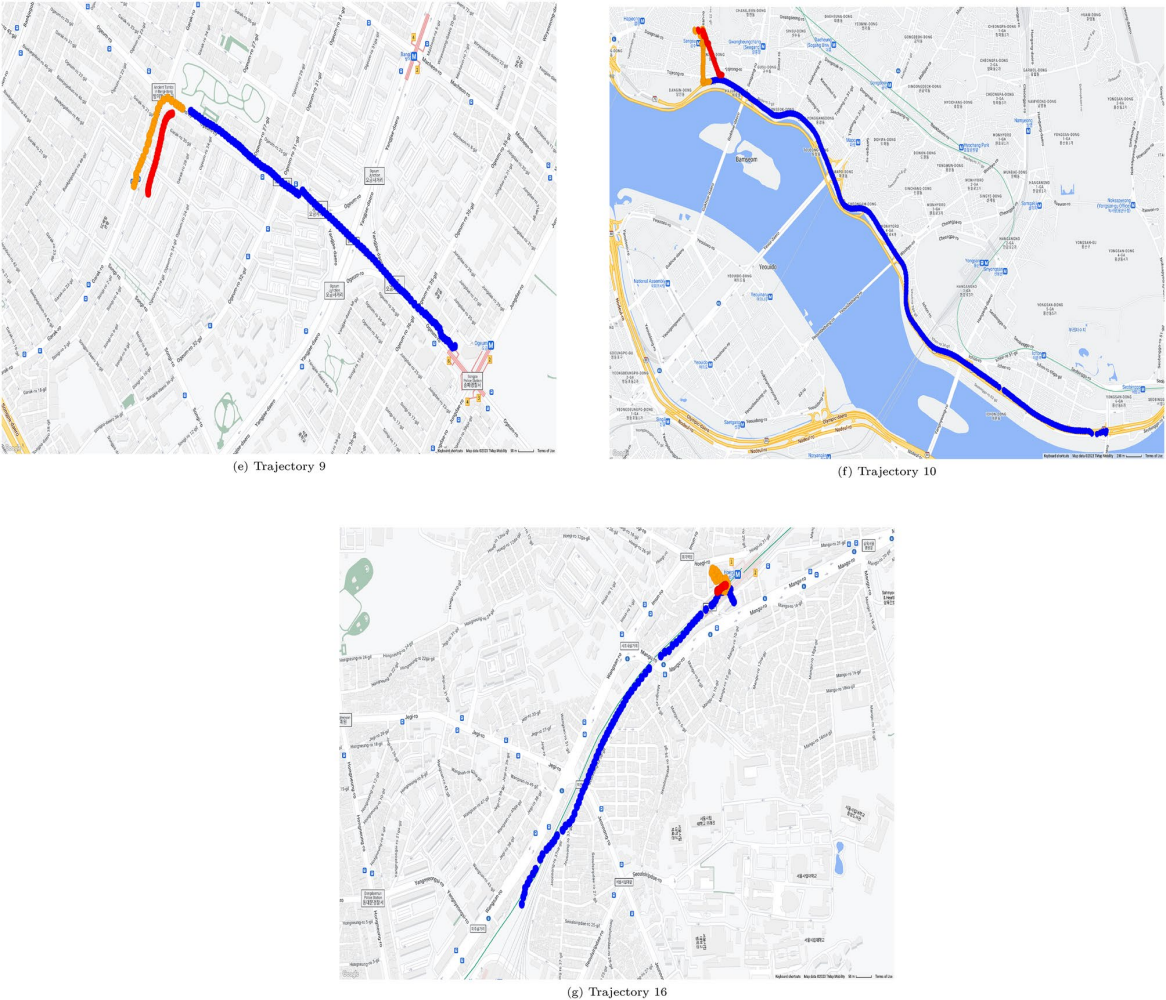
Prediction results mapped for C type data set.

**Figure 6:**
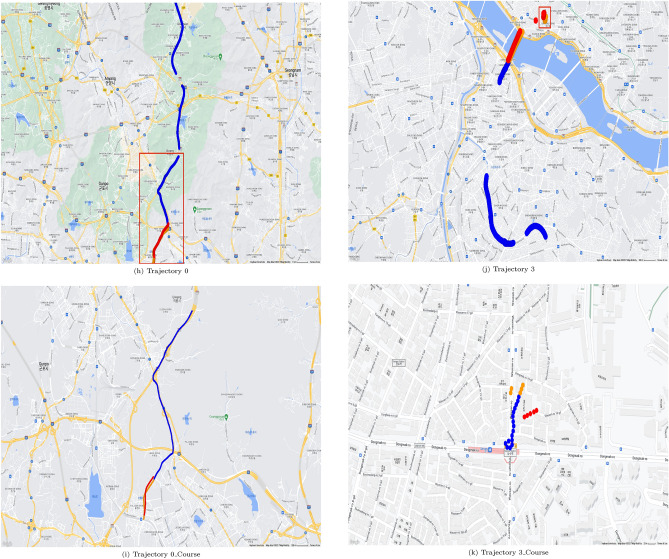

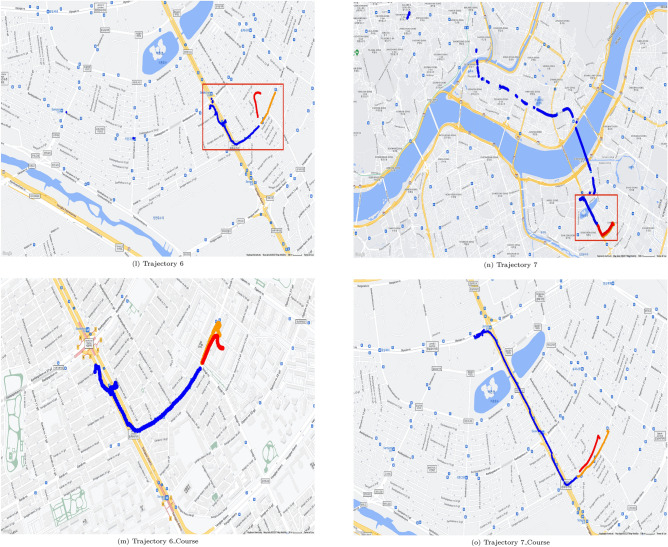

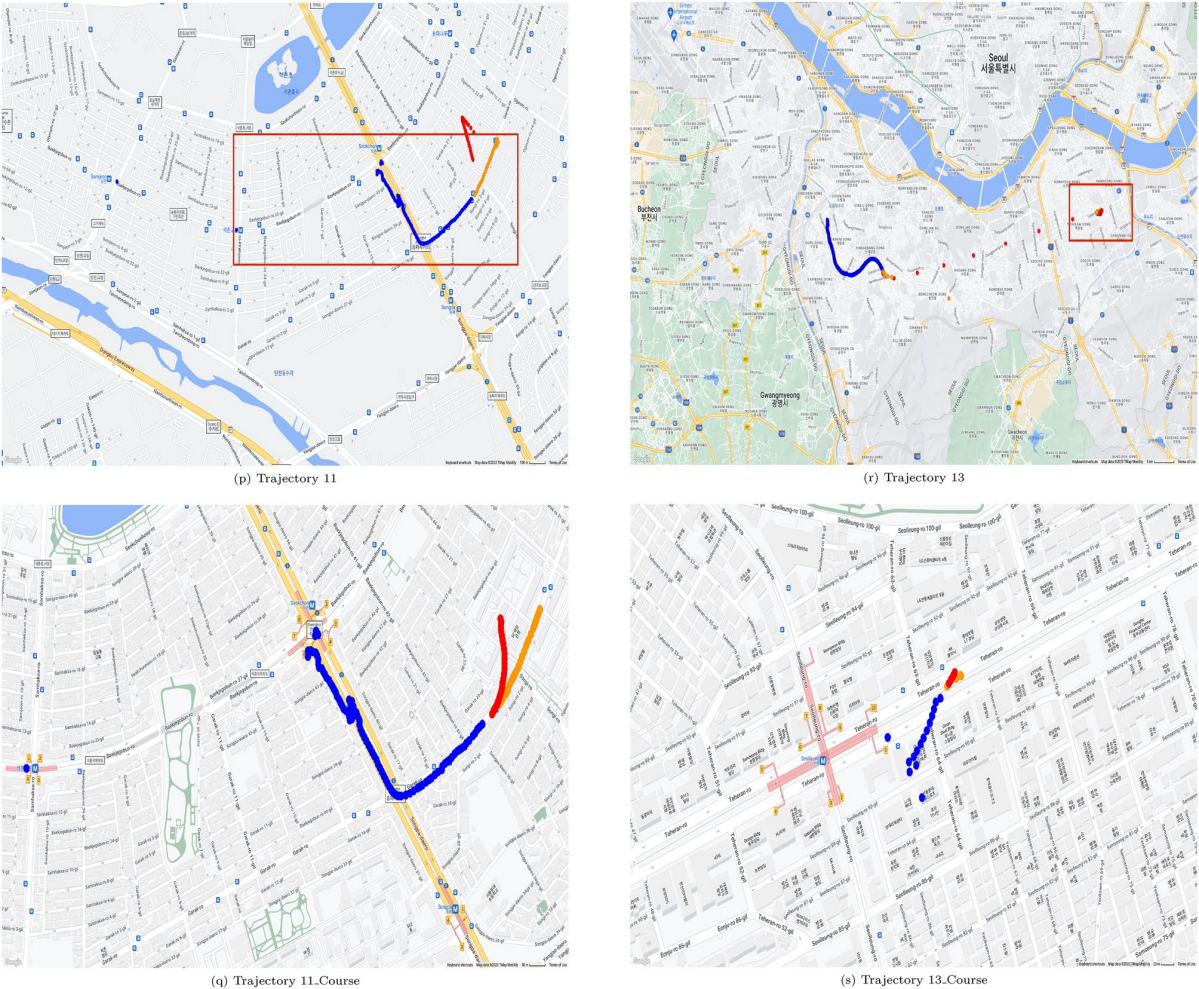
Prediction results mapped for N type data set.

Figures 5 and 6 show geopositioning data in actual maps. These figures were generated by use of gmplot^18^ which is a python library for using google map api, and google map permits free use for the purpose of research paper writing^19^. The real position data of the training data are represented by blue dots in Figs. 5 and 6, the yellow parts represent the actual location data of the test data, and the red parts represent the values predicted through the test data. The outcome using C type data sets is shown in Fig. 5, and the outcome using N type data sets is shown in Fig. 6. It can be seen from Table 4 that when utilizing C type data sets, the resulting values for FDE, ADE, and training time are predictable. However, Table 5 reveals that the FDE, ADE, training time, and optimum Window_Size values are not predictable when using N type data sets. It can be seen that the model of this study is difficult to use with data cut in the middle.

## Treating non-continuous data sets

An efficient method to use N type data sets requires much consideration. The N type data sets were divided into C type data sets based on the broken parts. At this time, the C type data sets were defined as ‘course’ data sets. To distinguish them, the following process is required. The average of the distances was calculated after first determining the distance between each location. Subsequently, if the distance to the next location data was greater than the average, it was determined that the data was cut off. Through this process, the data set was partitioned. If there is a jump in distance between the 30th and 31st geopositioning data, and the distance between the 80th and 81st are greater than the average distance, 31st to 80th geopositioning data becomes a course data set. As the last course is C type data, the algorithm of this study can be applied.

A course data set has different training and verification sets from the original N type data set, although course data set is derived from a corresponding N type data set, because a course data set is simply a continuous part of an N type data set.

Examples are shown in Fig. 6h,i. Figure 6h shows training data set as blue dots, but the locations and the count of blue dots in Fig. 6i are different. In Fig. 6, for each N type data set, trajectory of N type data set and matched course pair of the same trajectory is shown. For each trajectory of N type data set, a red box shows course part. Then for each trajectory of N type data set, trajectory of course part only is shown.

**Table 8:** Results on course data selected from N type data.

Data	FDE (m)	ADE (m)	Training time (s)	Window size	Numer of data
0	162.904	197.703	10.480686	11	470
3	42.529	41.927	6.326526	2	23
6	202.317	205.214	4.184149	7	149
7	147.794	95.526	6.837206	10	240
11	147.314	119.618	7.119140	16	143
13	50.255	42.505	4.233325	7	25

Table 8 is the result of making C type data by tuning N type data into course data and running it through our algorithm. Because the number of data was reduced compared to Table 5, the training time was greatly reduced. Furthermore, the window size changed because it became a course data set. When compared to before the change to course data, the FDE and ADE values have evidently improved. An N type data set can be processed in this way by being converted into course data sets. This allows for the processing of N type data with only simple additional processing.

Course data sets are able to predict more accurately than N type data sets and can be visualized as shown in Fig. 6l versus Fig 6m,n versus Fig. 6o,p versus Fig. 6q. This approach was not that effective for Data Sets 3 and 13, which have relatively small data counts of 23 and 25 respectively, similar to the L type data set. With too small data count in a data set, it is almost impossible to apply any existing methods for location prediction.

**Table 9:** Comparison of results when using CPU and GPU using C type data.

Data set ID	Training time using CPU (s)	Window_Size	Training time using GPU (s)	Window_Size
1	7.459214	9	3.796056	5
2	8.530682	6	3.865496	7
4	14.810614	7	3.613486	7
8	64.190169	2	29.131446	2
9	8.243129	12	4.889933	15
10	14.457857	10	4.315538	6
16	6.678380	12	3.659727	12
Average time	17.767149		7.610241

**Table 10:** Comparison of results when using CPU and GPU using N type data.

Data set ID	Training time using CPU (s)	Window_Size	Training time using GPU (s)	Window_Size
0	18.205739	11	14.950944	12
3	7.532523	4	4.358422	4
6	6.347854	10	3.956954	11
7	8.200427	5	4.927039	6
11	8.945682	15	3.632642	17
13	23.193552	3	5.511075	6
Average time	12.070963		6.222846	

## Conclusion and future works

This study attempted to develop a system that uses IoT devices to gather location data and forecasts future locations using those data. We aimed to lower the amount of computing and reduce the training time because it is vital to be able to anticipate even with resource-restricted devices. Therefore, the algorithm should be simplified as much as possible, so that the accuracy is maximized, and the training time is reduced, to make it suitable for use on AIoT or EdgeML devices.

To drastically reduce the learning time, a GPU or an NPU can be used to shorten the training time further.

Tables 9 and 10 compare C and N type data sets when a CPU and GPU are used, respectively. Expectedly, results measured on a GPU show far smaller training time. The CPU used was an Intel(R) Core(TM) i5-8365 CPU@ 1.60 GHz, and the GPU was GPU T4 provided by Google Collaboratory (Colab)^17^. Once the training time is a critical point, it is recommended to use a GPU or an NPU because training time on a GPU was as fast as less than 4 s.

Table 11 compares the ADE and FDE of our model using CPU and GPU with the results from other methods. We chose two previous research results for comparison. An attention network was added and implemented in^9^, and an LSTM with social pooling was implemented in^8^. The ADE value is 0.5 and 0.27 respectively for other techniques, while our ADE value is 0.06; FDE is 1.06 and 0.61 respectively for other techniques, while our FDE is 0.07.

The ADE and FDE values were similar when we used a GPU in our model considering round off of values. However, in terms of training time, a CPU required 17.8 s, while a GPU took 7.6 s, which is overwhelmingly fast. Therefore, if we use a GPU in our model, we can achieve good results in terms of accuracy and time.

**Table 11:** Comparison with other methods focusing on ADE and FDE.

	ADE (m)	FDE (m)
Attention network	500	1060
Social network	270	610
Our model with CPU	60	70
Our model with GPU	60	70

**Table 12:** Comparison with other methods focusing on average training time.

	Average training time (s)	Window_Size
LSTM(look_back=10)	40.8642	10
LSTM(look_back=30)	51.6841	30
Our model with CPU	17.7671	Optimized_Window_Size
Our model with GPU	7.6102	Optimized_Window_Size

The average training time for our model and other strategies^10^ is compared in Table 12. A machine equipped with Intel(R) Core(TM) i7-7700 CPU@ 3.60 GHz, 32 GB RAM with GeForce GTX 1080 Ti GPU is used for the experiments described in^10^. For these experiments, we used Intel(R) Xeon(R) CPU @ 2.20 GHz and NVIDIA Tesla T4 provided by Google Colaboratory (Colab)^17^. An average of 45 s were needed to train the data; however, in our experiment, it was 17.7671 s when only a CPU was used, and 7.6102 s when a GPU was used. These results were acquired from a non-identical experimental environment. We cannot use the same GPU for comparison with the results from^10^.

However, we can conclude that our method is better than those described in^10^ in terms of training time, ADE, and FDE values because we introduced optimization of the window size hyperparameter. We used CPU and GPU, and our result with CPU only shows better training time than that of comparison target.

From the results, we can conclude that we obtained better experimental results compared with previous research results, especially for C type data sets. In the case of N type data sets, we had less accuracy, with 594m for ADE and 585m for FDE. To solve these phenomena, we introduced the concept of course data. We cut the very final part of an N type data set so that the final part is guaranteed to be a continuous geopositioning data set. Applying our method again to course data sets, we obtained significantly better results in terms of accuracy and training time, as shown in Section “Treating non-continuous data sets”. However, with data sets with very low data counts, we were unable to achieve better results, similar to the case of L type data sets. It can be seen that the method used for this study works effectively when the data is reliable and consists of an adequate count of geopositioning data. Another mechanism to fill the lost data is therefore required. A further study will possibly address these issues utilizing a generative adversarial network (GAN)^20^.

The purpose of this research is to present a simple and lightweight method which could be applicable to lower computing capability devices, such as AIoT or EdgeML devices. This makes it suitable for use in the Third World, where devices and networks are scarce. Providing location prediction services at low computing power will enable people in Third World countries utilize the location-based services we currently use. Furthermore, it would be more useful to use the service in places such as over the countryside, for example in a military conflict, rather than in a general situation or location. In such a scenario, individual solders can carry these devices, predict the movement trajectory of adversaries, and efficiently mount counter-offensive operations.

## Data Availability

The raw datasets used and analyzed during the current study available from the corresponding author on reasonable request.

## References

[1] Ververidis, C., Polyzos, G. Mobile marketing using a location based service. In *Proceedings of the First International Conference on Mobile Business*, 1– 12 (Athens, Greece, 2002).

[2] Budjac, R., Barton, M., Schreiber, P., Skovajsa, M. Analyzing embedded AIOT devices for deep learning purposes. In *Computer Science On-line Conference*, 434–448 (Springer, 2022).

[3] Zhao, Z., Wang, K., Ling, N., Xing, G.: Edgeml: An automl framework for real-time deep learning on the edge. In *Proceedings of the International Conference on Internet-of-Things Design and Implementation*, 133– 144 (2021).

[4] Yu Y, Si X, Hu C, Zhang J (2019). A review of recurrent neural networks: LSTM cells and network architectures. Neural Comput..

[5] You D, Song HY (2020). Trajectory pattern construction and next location prediction of individual human mobility with deep learning models. J. Comput. Sci. Eng..

[6] Pascanu, R., Mikolov, T., Bengio, Y.: On the difficulty of training recurrent neural networks. In *International Conference on Machine Learning* 1310– 1318 (PMLR, 2013).

[7] Manaswi, N. K. RNN and LSTM, 115– 126 (Apress, Berkeley, CA, 2018). 10.1007/978-1-4842-3516-4_9.

[8] Alahi, A., Goel, K., Ramanathan, V., Robicquet, A., Fei-Fei, L., Savarese, S. Social LSTM: Human trajectory prediction in crowded spaces. In *Proceedings of the IEEE Conference on Computer Vision and Pattern Recognition*, 961– 971 (2016).

[9] Sim, S., Min, J., Kim, B., Kim, J.: *Pedestrian Trajectory Prediction Using Attention Network*. The Institute of Electronics and Information Engineers, 552–555 (2020).

[10] Yoon S-W, Lee W-H, Lee K-C (2022). Pedestrian GPS trajectory prediction deep learning model and method. J. Korea Soc. Comput. Inf..

[11] Muraina, I. Ideal dataset splitting ratios in machine learning algorithms: General concerns for data scientists and data analysts. In *7th International Mardin Artuklu Scientific Research Conference* (2022).

[12] Torgerson WS (1958). Theory and Methods of Scaling.

[13] Yang L, Shami A (2020). On hyperparameter optimization of machine learning algorithms: Theory and practice. Neurocomputing.

[14] Kuusk A, Kuusk J, Lang M (2009). A dataset for the validation of reflectance models. Remote Sens. Environ..

[15] Sinnott, R. W. Virtues of the haversine. *Sky and Telescope* **68**(2), 158 (1984).

[16] Dauni P, Firdaus MD, Asfariani R, Saputra MIN, Hidayat AA, Zulfikar WB (2019). Implementation of haversine formula for school location tracking. J. Phys. Conf. Ser..

[17] Nelson, M. J., Hoover, A. K.: Notes on using google colaboratory in ai education. In *Proceedings of the 2020 ACM Conference on Innovation and Technology in Computer Science Education. ITiCSE ’20*, 533– 34. Association for Computing Machinery, New York, NY, USA (2020). 10.1145/3341525.3393997.

[18] https://github.com/gmplot/gmplot.

[19] https://www.google.com/intl/en-GB_ALL/permissions/geoguidelines/.

[20] Liang, X., Hu, Z., Zhang, H., Gan, C., Xing, E. P. Recurrent topic-transition GAN for visual paragraph generation. In *Proceedings of the IEEE International Conference on Computer Vision (ICCV)* (2017).

